# Electrochemical generation of microbubbles by carbon nanotube interdigital electrodes to increase the permeability and material uptakes of cancer cells

**DOI:** 10.1080/10717544.2019.1662514

**Published:** 2019-09-16

**Authors:** Mohammad Ali Khayamian, Shahriar Shalileh, Shohreh Vanaei, Mohammad Salemizadeh Parizi, Saeid Ansaryan, Mohammad Saghafi, Fereshteh Abbasvandi, Amirali Ebadi, Pouya Soltan Khamsi, Mohammad Abdolahad

**Affiliations:** a Nano Electronic Center of Excellence, Nano Bio Electronic Devices Lab, School of Electrical and Computer Engineering, University of Tehran, Tehran, Iran;; b Nano Electronic Center of Excellence, Thin Film and Nanoelectronic Lab, School of Electrical and Computer Engineering, University of Tehran, Tehran, Iran;; c School of Mechanical Engineering, College of Engineering, University of Tehran, Tehran, Iran;; d School of Biology, College of Science, University of Tehran, Tehran, Iran;; e ATMP Department, Breast Cancer Research Center, Motamed Cancer Institute, ACECR, Tehran, Iran;; f MEMS and NEMS Laboratory, Department of Electrical and Computer Engineering, Faculty of Engineering, University of Tehran, Tehran, Iran

**Keywords:** Microbubble generation, electrochemistry, sonoporation, ultrasonic stimulation, drug delivery

## Abstract

Artificial cavitation as a prerequisite of sonoporation, plays an important role on the ultrasound (US) assisted drug delivery systems. In this study, we have proposed a new method of microbubble (MB) generation by local electrolysis of the medium. An integrated interdigital array of three-electrode system was designed and patterned on a nickel-coated quartz substrate and then, a short DC electrical pulse was applied that consequently resulted in distributed generation of microbubbles at the periphery of the electrodes. Growth of the carbon nanotube (CNT) nanostructures on the surface of the electrodes approximately increased the number of generated microbubbles up to 7-fold and decreased their average size from ∼20 µm for bare to ∼7 µm for CNT electrodes. After optimizing the three-electrode system, biocompatibility assays of the CNT electrodes stimulated by DC electrical micropulses were conducted. Finally, the effect of the proposed method on the sonoporation efficiency and drug uptake of breast cells were assessed using cell cycle and Annexin V/PI flow cytometry analysis. These results show the potential of electrochemical generation of MBs by CNT electrodes as an easy, available and promising technique for artificial cavitation and ultrasound assisted drug delivery.

## 1. Introduction

Cancer has gathered the scientists around the world for propounding an idea to harness one of the very mortal diseases of the current century. Chemotherapy or delivery of anticancer drugs to the tumorigenic tissue has been a conventional method of cancer therapy (Skeel & Khleif, 2011; Wagner et al., 2017), which has been always accompanied with some challenges such as chemo-resistance of the cancerous cells (Wang et al., 2010; Abdullah & Chow, 2013) and detrimental side effects of the designed drugs (Monsuez et al., 2010; Florea & Büsselberg, 2011). To reduce the side-effects, various drug delivery systems including photothermal therapy (Huang et al., 2006; Riley & Day, 2017), ultrasound-assisted chemotherapy (Couture et al., 2014; Mullick Chowdhury et al., 2017), alternating electric field therapy (Kim et al., 2016; Rick et al., 2018) etc. have been developed to stimulate the site of interest, which would subsequently enhance transport of chemotherapeutics to the malignant region with minimum damage to the other organs (Yao et al., 2016). Maximizing the drug uptake in targeted lesions along with minimizing the primary dose of drug not only would increase the treating efficacy but also would reduce the therapeutic side effects.

In this regard, selective enhancement of the permeability of cancer tumor to drug would be a great solution. Ultrasound-assisted drug-delivery systems which are based on the phenomena of sonoporation (Lentacker et al., 2014; Yu & Xu, 2014), has emerged as a promising method of cancer treatments with the benefits of low side effects as well as high efficiency (Mitragotri, 2005; Boissenot et al., 2016). Many methods have been developed on selective drug uptake in cancer lesion in which the drug entrance in sonoporated cells was more significant (Rapoport, 2004; Xing et al., 2016; Shen et al., 2017). Enriching the medium with polymeric commercial microbubbles is a conventional technique to better induce sonoporation in targeted cells and facilitate the transport of drugs and compounds into the cell by supplementing the number of microbubbles and the subsequent surge of sonoporation (Quaia, 2007; Unger et al., 2014). Recently, we introduced electrochemically active microneedles coated by ZnO nanowires for in-vivo generation of MBs from intercostal fluids and enhanced US assisted drug delivery to tumors in mice models (Zandi et al., 2019).

Here we applied similar in-situ generation of microbubbles with the assistance of Carbon nanotubes decorated on interdigital electrodes for electrochemical stimulation of the peripheral media to induce sonopores in MDA-MB-231 breast cell line. This in-vitro actuating biochip were interacted with anti-cancer drug Paclitaxel and the treating efficacy was compared with control samples. Effect of planar and interdigital electrodes in production and distribution of MBs were also compared.

## 2. Experimental setup

**Figure 1.**
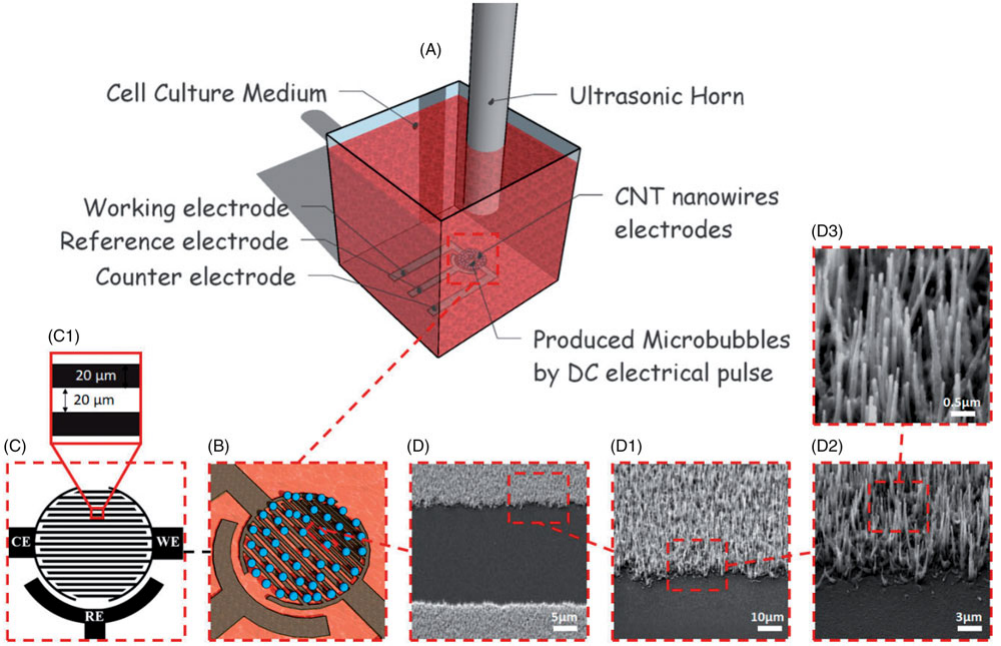
Schematic representation of the experiment (A). Scheme of generated microbubbles on the surface of the working and counter electrodes (B). Geometry and feature size of the interdigitated three-electrode system (C). SEM images of the grown CNT on the surface of the quartz as the substrate for the three-electrode structure (D).

As presented in Figure 1-A the set of three interdigital patterned working, reference and counter electrodes on the quartz substrate were placed at the bottom of a PMMA chamber and MDA-MB-231 breast cancer cells were seeded on its surface. After seeding, the chamber was filled with cell culture medium (89% DMEM, 10% FBS, and 1% antibiotics) containing desired concentration of the Paclitaxel (PTX) anticancer drug in different groups of treatment which served as the ionic electrolyte for local electrolysis of the solution. Before US stimulation, the microbubbles were generated by DC micropulses to the electrodes and after placing the US horn above the electrodes surface the ultrasound was applied.

### 2.1. Microbubble generation and US parameter specification

Before applying ultrasound, microbubbles were generated by applying a short DC electrical pulse to provide an artificial cavitation that could enhance the efficacy of sonoporation. In this regard, -1.3 V as the roughly minimum potential which causes medium electrolysis was extracted for both bare and CNT-grown electrodes. It is worth mentioning that the applied potential is the voltage between the working and reference electrode. This is an instantaneous potential which lasts for 1 mSec and gives a rise to the electrolysis of the medium at the interface of the working and counter electrodes (Figure 1-B).

Duration of 2 seconds, intensity of 1.8 W/cm^2^ and frequency of 20 KHz were selected as the parameters of ultrasound stimulation (Khayamian et al., 2018). The frequency and intensity of the US stimulation stands in the range of low frequency and low intensity, which is suitable and recommended for US drug delivery systems (Wood & Sehgal, 2015). In addition, 4 cm was the distance between the US horn and electrode’s surface (Khayamian et al., 2018).

### 2.2. Electrode fabrication and CNT nanowire growth

**Figure 2.**
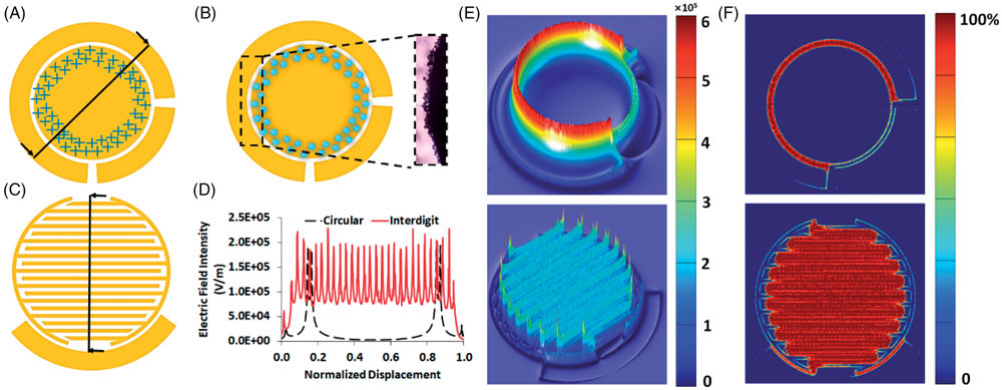
Charge accumulation on the priphery of the working electrode (A) and subsequent microbubble generation (B) in the typical circular three-electrode system. Structure and desgin of the interdigitated array of the electrodes (C). Comparison of the cross-sectional slice of the electric field distribution (D) as well as 3d plot (E) in the circular vs. interdigit three-electrode system. Contour plot of 95–100% of maximum E-field in the circular and interdigit arrays (F).

Two arrays of circular and interdigitated three electrode systems were used in order to compare the efficiency of the microbubble generation. In the case of circular (Figure 2-A), the three-electrode system contains a circular working electrode (WE) with diameter of 5 mm which is surrounded by a 1 mm thickness of counter electrode (CE). Additionally, 20 µm is the distance between the working and counter electrodes. For interdigital array of the electrodes (Figure 1-C), the spacing between the electrodes as well as blades thickness are 20 µm (Figure 1-C1).

The CNT growth process on the electrodes of both circular and interdigital arrays, starts by depositing a thin film of Ni as a catalyst on quartz surface followed by patterning the designed arrays of the electrodes using standard photolithography system. Finally, the sample is placed in a direct-current plasma enhanced chemical vapor deposition (DC-PECVD) reactor to grow vertically aligned multi-walled carbon nanotubes (MWCNT) on Ni-deposited regions in a manner reported elsewhere (Abdi et al., 2007; Abdolahad et al., 2012). FESEM graphs in Figure 1-D reveals the high-quality growth of vertical CNT nanowires over the electrodes with desired pattern and geometry.

### 2.3. Drug dosage determination by MTT assay

MTT assay was used to determine the effect of different dosages of PTX on the viability of MDA-MB-231 cells after 24 hours. At first, the cells with 70% confluency was cultured in 96 well-plate and then the bare medium was replaced with a medium fresh medium containing PTX anticancer drug with different concentrations. After 24 hours, 20 µl 3-(4,5-Dimethylthiazol-2-Yl)-2,5-Diphenyltetrazolium Bromide (MTT) (50 µg/ml) was added to the medium and incubated for 4 hours. Next, the medium was removed and substituted with 200 µl of DMSO (Dimethyl sulfoxide) and after 5 minutes of shaking the absorbance of the colored solution was quantified by a spectrophotometer (BioTek, Winooski, VT, USA) at 570 nm wavelength (Mosmann, 1983).

### 2.4. Annexin V/PI viability assay

Percentage of the dead vs living cells in treatment groups were investigated by Annexin V/PI (AV/PI) viability assay (Pan et al., 2007). To do this, after the end of treatment the cells were collected and resuspended in a 500 µl of binding buffer. In the next step, 5 µl of PI dye and Annexin V-FITC were added to the cell suspension and incubated for 10 minutes in dark at room temperature. Next, the cells were transferred to the flow cytometer (FACScan Becton Dickinson, Mountain View, CA) and the results were evaluated.

### 2.5. Cell cycle flow cytometry assay

In order to analyze the cell phase in different groups of treatment, cell cycle flow cytometry was carried out. When the desired treatment was finished cell of different groups were trypsinized and detached from the electrodes and gently fixed by 70% chilled ethanol. Then, propidium iodide (PI) dye with the concentration of 20 µg/ml was added to the solution and incubated 10 minutes in a dark room. Prior to this step and due to capability of PI binding to RNA, RNase solution is used to remove free RNA residues and decrease the nondesired attachment of the PI to RNA. At the end, the PI fluorescence was analyzed by a flow cytometer (FACScan Becton Dickinson, Mountain View, CA).

### 2.6. Immunofluorescence confocal microscopy

To evaluate the incidence of sonoporation as well as influence of various groups of treatment on the cell cytoskeleton structure, confocal microscopy of F-actin microfilaments and microtubule components was applied. After each treatment, the cells were fixed using 3.7% paraformaldehyde followed by using 0.1% Triton-X100 diluted in PBS as permeabilizing agent, both for 10 minutes. Thereafter, the blocking step was performed for 30 minutes by PBS containing 1% BSA. Primary microtubule antibody (Alpha Tubulin Monoclonal Antibody, Invitrogen) was added to the cells for 3 hours and then the secondary agent (Goat anti-mouse secondary antibody conjugated with Alexa Fluor 555, Invitrogen) was applied for 1 hour. After microtubule staining, actin staining dye (Alexa Fluor® 488 Phalloidin, Invitrogen) was added to the cells and incubated for 20 minutes. Finally, and after mounting on a glass slide (ProLong^®^ Diamond Antifade Mountants, Invitrogen), the cells were imaged by confocal microscope system (Leica, TCS SP5, Germany).

### 2.7. Field emission scanning electron microscopy (FESEM)

To pursue the trace of the sonoporation on the cells induced by the generated microbubbles, scanning electron microscopy was utilized. After microbubble generation and the following US stimulation, the medium was removed and cells were immediately fixed by glutaraldehyde 5% for 5 minutes. After triplicate washing with PBS, the cells were dipped into distilled water and immediately dried by air. Then, the cells were gold sputtered for 500 s and then transferred into the FESEM chamber (Hitachi-S4160).

### 2.8. Cell culture

MDA-MB-231 metastatic cancer cell line as the most drug resistant line of the human breast cancer (ref) were purchased from National Cell Bank of Pasteur Institute of Iran. The cells were cultured in Dulbecco's Modified Eagle's Medium (DMEM, Sigma) containing 10% fetal bovine serum (FBS, Gibco) and 1% penicillin/streptomycin (PenStrep, Gibco) and maintained under standard cell culturing conditions (37 °C, 5% CO_2_). Every two days the cell culture medium was replaced with fresh one.

## 3. Results and discussion

### 3.1. Electrode design, fabrication and characterization

MB generation was carried out using conventional electrochemical system encompassing counter (CE), reference (RE) and working (WE) electrodes (Figure 2A and C). The RE senses the potential of the solution and the voltage of WE is set relative to the RE. Also, the current is established between counter and working electrode. A short DC electrical pulse (potential of -2 V and pulse duration of 1 ms) was applied to electrolyze the cell culture media as the background solution and this causes generation of MBs, which facilitates the sonoporation and increases the efficiency of the drug delivery.

A typical array of the system is a circular shape of the working electrode and a counter electrode that surrounds it (Figure 2-A). Strong electric field at the edge of WEs (named as edge effect (Resnick et al., 1988)) resulted in production of MBs with higher concentrations at those regions (Figure 2-B). Based on this phenomenon, distribution of the edges all over the surface of the WE could result in produced MBs with uniform distribution on the surface (Figure 2-C). Hence, an interdigitated working electrode would be better than planar metal electrode.

To assess how the electric field is distributed in the both arrays of the working electrodes (circular vs interdigitated) COMSOL Multiphysics finite element software (AC/DC electrostatic module) was utilized. The working potential was swept to reach the minimum threshold of inducing electrolysis and MBs in the solution. -1.4 V was the experimentally selected voltage. The reference potential was set to zero and the counter potential was set to +1.2 V. As presented at the Figure 2-D and -E, the cross-sectional slice of the electric field distribution in the both systems indicate the E-field was maximized at the edge of the blades. Presence of more sharp peaks as the responsible points that generate microbubbles, confirm that the uniformity of the MBs and the sites of bubble generation could be amplified in IDT conformation. In addition, Figure 2-F displays a contour plot of 95-100% of maximum E-field in the both IDT and planar electrodes. The results demonstrate that the E-field decreases suddenly in the planar array whereas stays stable and uniform with a tiny weakening in strength across the blades in IDT pattern (Figure 2-F). This corroborates that the IDT array distributes the E-field uniformly all over the surface of the WE, which brings about the consequent uniformity in the generation of microbubbles.

**Figure 3.**
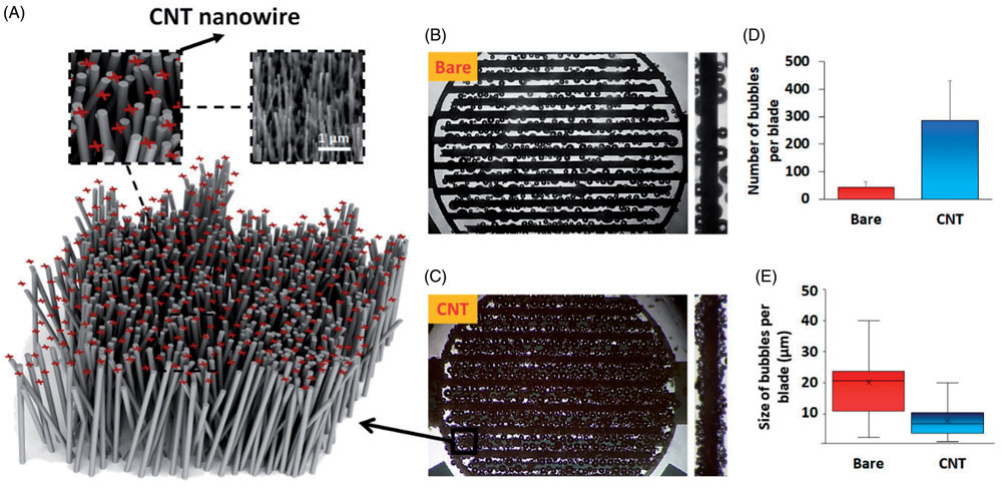
Scheme of the charge accumulation on the sharp tips of the carbon nanotube structures (A). Microbubble generation on the surface of the bare (B) and CNT-grown (C) interdigit array. Comparing number (D) and size (E) of bubbles per blade for bare and CNT-decoaretd interdigit array.

CNTs with thousands of dense sharp tips could upsurge the number of MB generation sites due to accumulation of the charge on the tips of the nanotubes and amplification of the E-field (Figure 3-A). Therefore, superimpose of CNTs on the IDT array electrodes offers the best-optimized electrode for microbubble generation. Figure 3-B and -C display the microbubbles generated at the bare and CNT-grown electrodes after applying short DC electrical pulse. As could be seen, the number of produced microbubbles (Figure 3-D) on the surface of the CNT WE is much higher (∼7 times) comparing to the bare one. In addition to the number, size of the microbubbles were also decreased from ∼20 in bare to ∼7 in CNT-grown IDT electrodes (Figure 3-E). This reduction in the size of the microbubbles could be owing to the dense proximity of the sharp tips of CNT decorated WE and RE as the responsible sites of microbubble generation, which does not allow the microbubbles to be expanded in an uncontrolled way.

### 3.2. Tracking biological impact of electrodes and produced MBs on the cells

**Figure 4.**
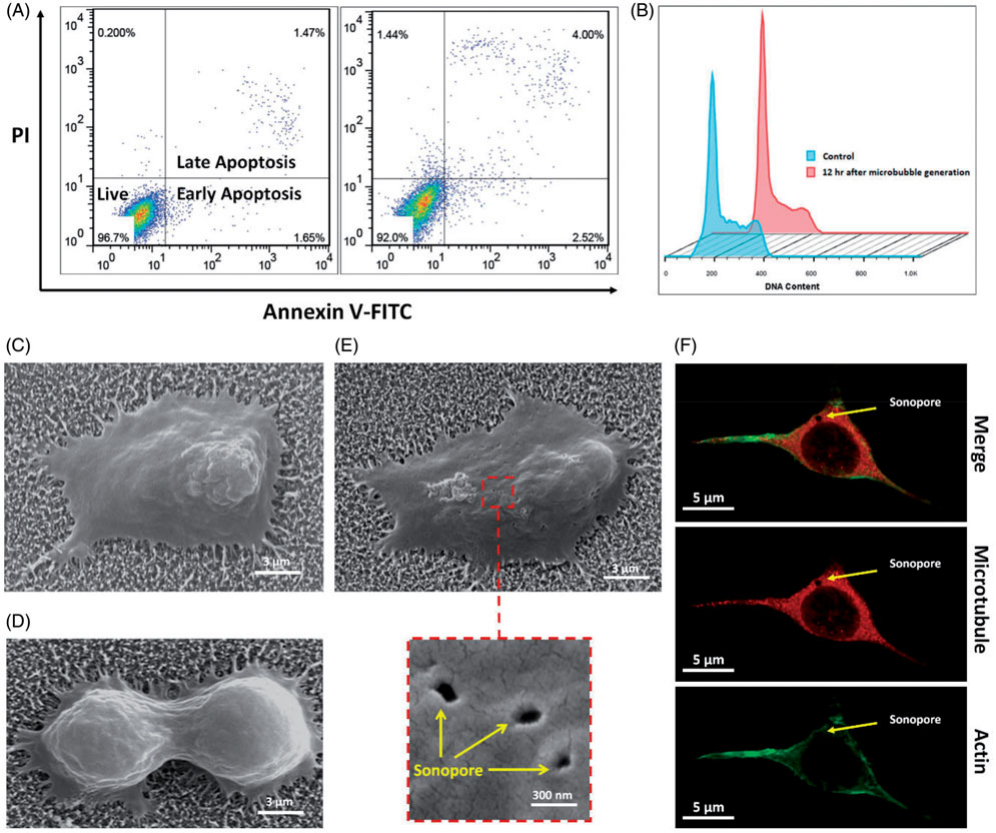
Annexin V/PI (A) and cell cycle (B) flow cytometry assays for the control and cells cultured on CNT and stmulated by short electrical pulses and subsequent MB generation. Spread (C) and mitosis (D) of the cancer cells cultured on the CNT nanostructures. Trace of the sonoporation on the surface of the US stimulated cancer cells (E). Confocal microscopy from a sonoporated cell (F).

Biocompatibility and safety of applying short electrical pulses and subsequent generation of microbubbles as well as using CNT nanostructures on the cultured cells were investigated by AV/PI and cell cycle flow cytometry assays. As presented in Figure 4-A, the comparative viability assays were conducted between control and two groups of individual treatments: cells seeded on CNTs and stimulated with electrical micropulse. After incubating overnight on the CNT substrate, the cells were detached from the substrate and analyzed by AV/PI analysis. The results indicate no signs of further death in the cells seeded on the CNT vs control group. Also, 12 hours after MBs generation, the cells seeded on the bare electrodes were assayed in similar manner and similar viability were observed. Moreover, cell cycle flow cytometry results from the groups of cells cultured on the CNTs before and 12 hours after applying short electrical pulse and generation of microbubbles, demonstrated no specific phase arrest meanwhile the life cycle of the cells has not altered (Figure 4-B). In addition, FESEM images from the cells cultured on the CNT substrate was carried out and a well spread (Figure 4-C) and mitosis (Figure 4-D) of the cells on the CNT nanostructured electrodes could be detected, respectively.

To track the trace of sonoporation on the cells induced by electrically generated microbubbles, cells cultured on CNTs and stimulated by electric pulses were exposed to ultrasound and immediately afterwards were fixed and analyzed by FESEM. As could be seen in Figure 4-E, the signs of sonoporation due to produced microbubbles could be traced on the membrane. Moreover, confocal microscopy was also conducted which indicated the trace of the sonoporation on the cell’s cytoskeleton and confirms the non-superficial effect of the sonopores and microbubbles (Figure 4-F).

### 3.3. Effect of generated MBs on material uptake of the cells

To reveal the true effect of MB generation on US assisted cellular uptake, PTX was choosed as the material for uptake and the cellular viability and proliferation, might be induced by PTX, was suggested as indication for level of uptake. 5 groups of treatments were considered:

1. Control: as cultured MD-MB-231 cells without US stimulation, PTX and MB generation.
2. US + MBs: to evaluate the sole effect of US stimulation and MB generation on the cells.
3. PTX: no ultrasound and microbubbles are used.
4. US + PTX: no microbubble generation is performed.
5. US + PTX + MBs: in addition to drug administration, ultrasound stimulation as well as microbubble generation is applied.

US parameters were set as mentioned in materials and methods section, and the IC_20_ (1.56 µg/ml) of the paclitaxel was selected as drug dosage which was extracted by MTT assay. The optimum potentials applied to the electrodes for electrolysis of the cell culture medium were obtained as discussed previously and -1.3 V was selected as the voltage of working electrode.

**Figure 5.**
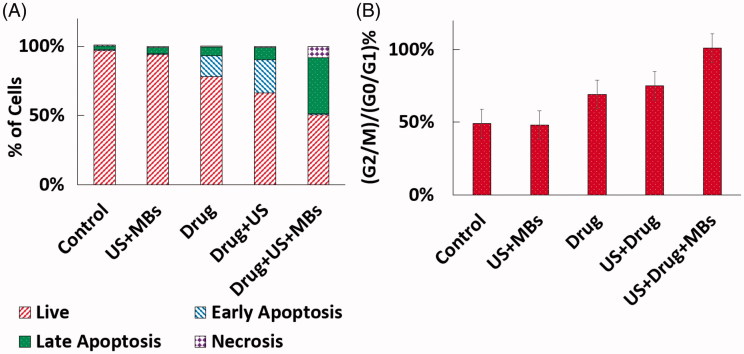
Viability results of the different treatments in the 5 groups obtained by Annexin V/PI flow cytometry analysis (A). The ratio of G2/M to G0/G1 peaks in the five regimes of treatment analyzed by cell cycle flow cytometry method (B).

Figure S1-A and Figure 5-A presents the viability results of the different treatment in the 5 groups acquired by Annexin V/PI flow cytometry analysis. As it could be deduced from the Figure 5-A, the number of dead cells is in harmony with the strength of the treatment. For instance, the results show substantial decrease of the living cancer cells when US is added to the drug treatment (Drug + US vs Drug group) and in a similar way, this number hit a low when the microbubbles are generated prior to the US stimulation (US + Drug + MBs). Comparing the effective number of dead cells in the last two groups (see supplementary Table S1) gives the effectiveness of using generated microbubbles. The result confirms that the generated microbubbles surges the sonoporation and the efficacy of cellular PTX uptake up to nearly ∼140%.

In addition to AV/PI viability assay, cell cycle flow cytometry assay was conducted to evaluate the impact of the different treatments on the cell cycle phases of the cancer cells. When a cell is entered into the mitosis phase of its life cycle, the transition from metaphase to anaphase occurs by depolymerization of the microtubules originated from the centrosomes to the chromosomes (Mitchison, 1971). Nevertheless, since the paclitaxel prevents depolymerization of microtubules, therefore the cell is not able to enter to the anaphase and is remained at metaphase stage (Schiff & Horwitz, 1980; Weaver, 2014). Hence, cells are arrested in mitotic phase and consequently could be tracked in the cell cycle flow cytometry. Figure S1-B demonstrates the different cell cycle phase of MDA-MB-231 cells in the 5 groups of treatment. As expected, the groups of control and US + MBs indicate no mitotic arrest in their typical graph of cell cycle, which pertains to lack of PTX uptake by the cells. The ratio of G_2_/M to G_0_/G_1_ peaks are displayed in the Figure 5-B. Fall in the peak of G_0_/G_1_ and rise of G_2_/M peak as the sign of mitosis arrest could be observed in the latter 3 groups of treatment which reaches a high in the US + Drug + MBs and corroborates the effect of generated microbubbles on better uptake of PTX by cells.

## 4. Conclusion

In order to increase the sonoporation efficacy, an artificial cavitation by electrolyzing the cell culture medium was carried out. A DC electrical micropulse was applied to an integrated three-electrode structure with an interdigital array. This IDT array caused the distribution of the electric field all over the surface of the electrodes and as a result, a uniform generation of microbubbles emerged. Growth of CNTs by providing thousands of sharp points throughout the surface of the electrodes offered a suitable platform to enhance the efficiency of the artificial cavitation. These nanostructures decreased the size of the microbubbles to ∼7 µm and increased their population to ∼7 times. This increase in the number and decrease in the MBs size lessens the risk of irreversibility as well as enhancing the efficiency of sonoporation, which eventually improves the material uptake by the cells. Cell cycle analysis and viability assays, corroborated the safety and non-toxicity of the CNT and short micropulses on the regular life cycle of the cells. Combining the proposed method of MB generation with ultrasound, we increased the PTX uptake in MDA-MB231 cells to ∼140% compared to the state that no microbubbles were generated. Therefore, the generated microbubbles could be a promising sonoporative agent for further studies in the fields of US assisted drug delivery as well as being applied as contrast agent for ultrasound imaging.
